# When the COVID-19 pandemic changed the follow-up landscape of chronic kidney disease: a survey of real-world nephrology practice

**DOI:** 10.1080/0886022X.2020.1798783

**Published:** 2020-07-28

**Authors:** Gang Chen, Yangzhong Zhou, Jinghua Xia, Jia Yao, Ke Zheng, Yan Qin, Xuemei Li

**Affiliations:** Department of Nephrology, Peking Union Medical College Hospital, Peking Union Medical College, Chinese Academy of Medical Sciences, Beijing, China

**Keywords:** COVID-19, chronic kidney disease, immunosuppressive treatment, follow-up, telemedicine

## Abstract

**Background:**

Patients with chronic kidney disease (CKD) require specialized management. However, the current situation of CKD management is unclear during the coronavirus disease 2019 (COVID-19) pandemic. We aimed to investigate the influence of the COVID-19 on kidney patients’ follow-ups.

**Methods:**

In April 2020, we included patients who underwent kidney biopsy from January 2017 to December 2019 in a referral center of China, and then initiated a survey *via* telephone on different aspects of follow-up during the COVID-19 pandemic. We collected and analyzed demographic data, diagnoses, follow-up conditions, and telemedicine experience.

**Results:**

We reached 1190 CKD patients with confirmed kidney biopsies, and included 1164 patients for analysis after excluding those on dialysis. None of our patients have had COVID-19, although more than 50% of them were complicated with other comorbidities, and over 40% were currently using immunosuppressive treatments. Face-to-face clinic visits were interrupted in 836 (71.82%) participants. Medicine adjustments and routine laboratory examinations were delayed or made irregular in about 60% of patients. To continue their follow-ups, 255 (21.90%) patients utilized telemedicine, and about 80% of them were satisfied with the experience. The proportion of telemedicine users was significantly higher in patients with immunosuppressive treatments than those without (31.88% vs. 17.12%, *p* < 0.001).

**Conclusion:**

The risk of COVID-19 was mitigated in patients with CKD and other co-existing risk factors when proper protection was utilized. The routine medical care was disrupted during the pandemic, and telemedicine could be a reasonable alternative method.

## Introduction

Since December 2019, the coronavirus disease 2019 (COVID-19) has developed into a global pandemic [1] and challenged the management of patients with chronic kidney diseases (CKD). First, kidney diseases require specialized management; these patients routinely visit clinics and require laboratory tests, and frequent medication adjustment is necessary for the newly diagnosed ones. However, the unprecedented social distancing measures inevitably disrupt their regular follow-up. The healthcare systems in many countries have reduced routine services, but such a strategy is not sustainable if the pandemic extends. Second, the COVID-19 death risk was predicted to be increased in patients featured with elder age and chronic comorbidities such as cardiovascular diseases [2–4]; such features were common in CKD patients [5]. However, data of COVID-19 incidence and effects of preventive measures in this population are lacking. Third, immunocompromise due to the corticosteroid and immunosuppressants is not uncommon in these patients. Nephrologists require reliable data to guide adjustment of the immunosuppressive treatment during this pandemic period. Last, the COVID-19 transmission could persist for months or years [6]; therefore, it may restructure our patient management now and in the future. A triage-in-nephrology protocol is in need.

Patients who have received kidney biopsies in recent years are a subset of CKD patients who require regular follow-ups. In countries with imbalanced medical resources, CKD patients routinely travel a long distance for regular follow-ups to some larger hospitals or specialized clinics. It would be valuable to learn from their follow-up experience during the pandemic period to rethink our follow-up management for patients with chronic diseases.

Peking Union Medical College Hospital (PUMCH) is a referral center located in Beijing, China. Over 70% of patients in the nephrology department come from other provinces, and most of the biopsied ones would prefer follow-ups in PUMCH despite the long distance. During the pandemic, the Chinese government has adopted massive public health interventions since late January 2020 to contain the viral spread. Several large cities have implemented strict quarantine, especially the epicenter of Wuhan, its neighboring provinces, and capital Beijing. The CKD follow-ups in PUMCH were inevitably affected during the first pandemic wave. In this study, we telephoned 1190 patients who have received kidney biopsy in the past three years and evaluated the effect of this pandemic on their disease management in the outpatient setting.

## Methods

We included all the patients who received kidney biopsy in the nephrology department of PUMCH from January 2017 to December 2019, then extracted necessary demographic data, diagnoses, and contact information from the medical records system. We telephoned the patients or their close relatives from April 15 to 25, to inquire about their follow-up conditions and telemedicine experiences during the COVID-19 pandemic. We excluded patients with inaccessible phone numbers and those who refused to participate or died before the pandemic. For evaluation of the follow-up conditions, we also excluded patients on renal replacement therapies, for they received their renal care in local dialysis units rather than in CKD clinics.

We inquired about follow-up conditions prior to the pandemic, on-going immunosuppressive treatments, and the influence of COVID-19 on follow-ups during the pandemic period. For previous follow-up conditions, the regular follow-up habit was defined as a generally uninterrupted face-to-face clinic visit with an interval of fewer than three months. We distinguished between PUMCH nephrology clinics and local hospitals for their previous hospitals for follow-ups. The on-going immunosuppressive treatments were categorized as steroid monotherapies, immunosuppressant monotherapies, and the combinations. We investigated the influence of COVID-19 on face-to-face clinic visits, laboratory examinations, medicine adjustments, medicine purchases, and the patients’ subjective perception of the clinic experience. For instance, we judged the laboratory examination as regular if it could be performed in the community health care facilities on time; the influence was considered minor if patients could timely adjust their medicine by following suggestions from local clinics or *via* telemedicine. For patients who experienced telemedicine, we investigated their communicating tools, starting time, and attitude toward telemedicine.

We used descriptive analysis to summarize the patients’ characteristics. The continuous variables were presented as mean ± standard deviation, while categorical variables summarized as a percentage. We used Pearson’s chi-square test to compare influencing factors between telemedicine users and non-telemedicine users. *p* < 0.05 was considered significantly different. All statistics were performed using the R program (Version 3.4.4, R core team).

## Results

**Figure 1.**
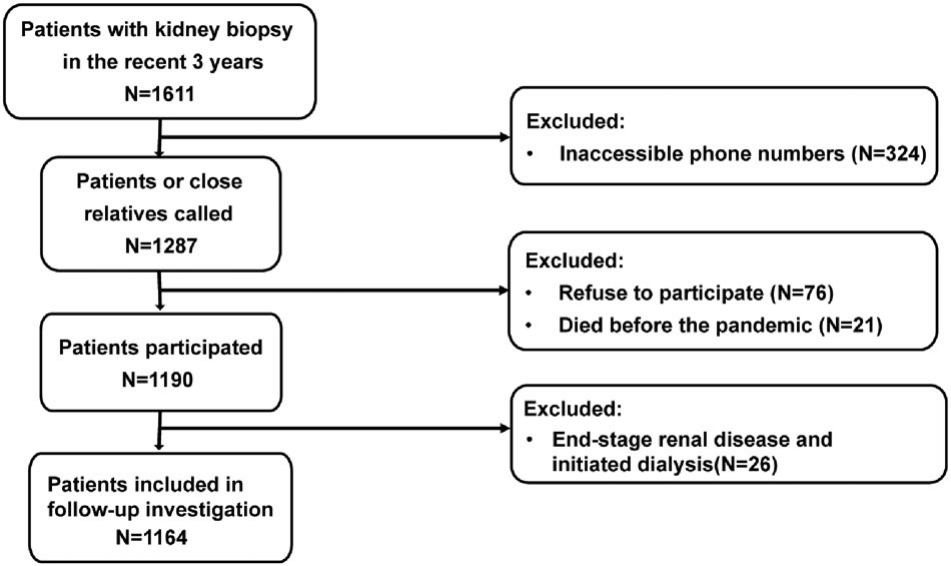
The flowchart of patient enrollment. We extracted demographic data, diagnoses, and contact information from 1611 patients with kidney diseases, whose kidney biopsies were performed from January 2017 to December 2019. We telephoned 1287 of them with available phone numbers from April 15 to 25. After excluding refusals and death, we reached 1190 patients in the end. We excluded 26 dialysis patients and included 1164 patients for analysis of follow-up conditions.

**Figure 2.**
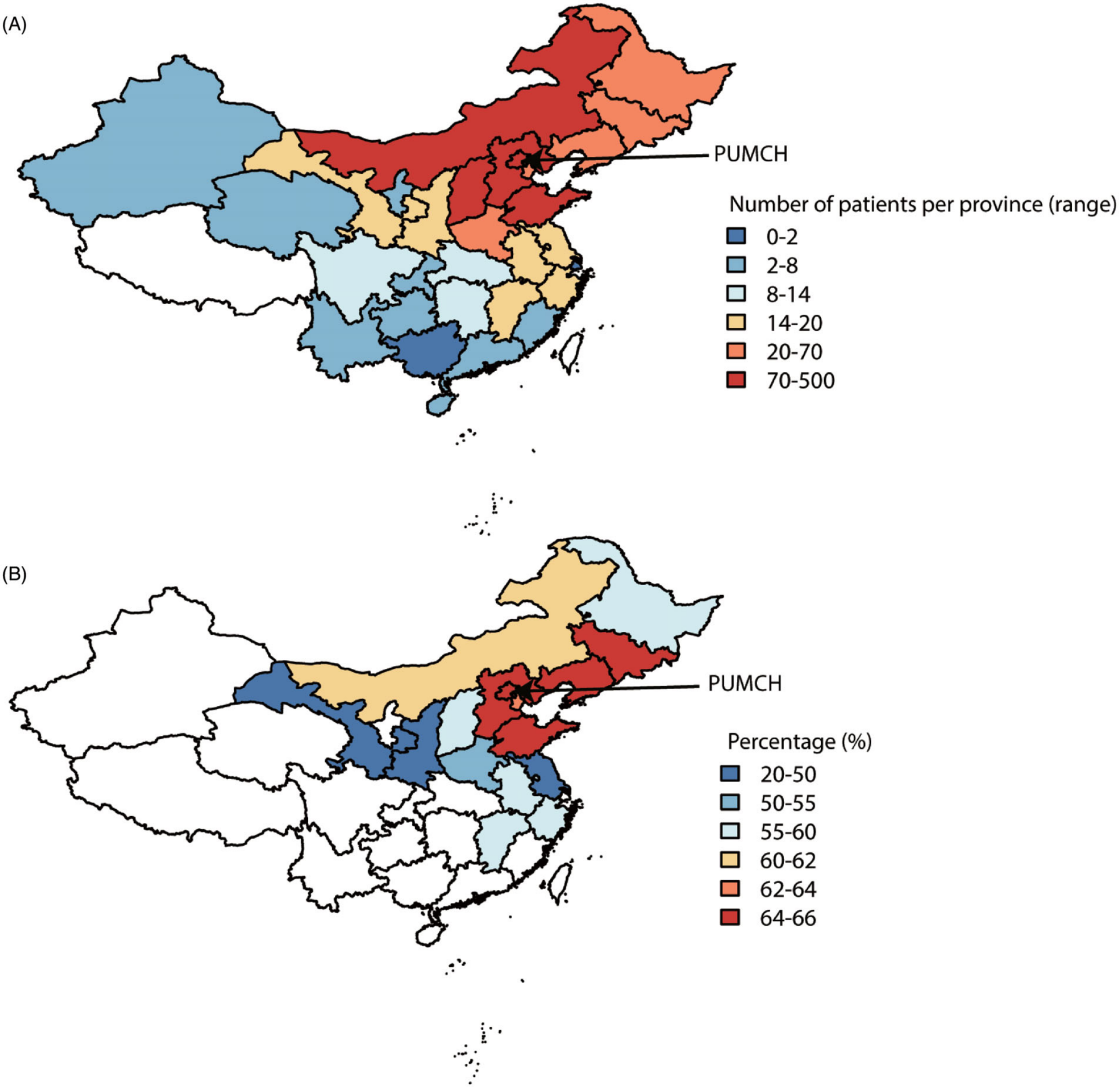
The geographic distribution of patients and follow-up conditions in China. (A) The patients enrolled in this study covered the major areas of China, especially the northeastern part. Peking Union Medical College Hospital is located in Beijing, where the most strict social distancing measures were adopted. Most of our patients were referred from surrounding areas. (B) The percentages of patients reporting interrupted follow-up differed between provinces, and only the provinces with more the ten patients were included in the analysis. Over 60% of patients were affected mainly in Beijing and surrounding provinces.

A total of 1611 CKD patients were documented in our medical record database from January 2017 to December 2019, whose diagnoses were confirmed by kidney biopsies. By April 25, 2020, we reached 1190 of them after ruling out those with inaccessible phone numbers (324) and patients who refused to participate (76) or died before the pandemic (21) (Figure 1). The average age of our patients was 41.91 ± 15.56 years old, and 52.10% were male. The median time since kidney biopsy was 23.62 (interquartile range [IQR] 3.72–40.19) months. Among included patients, 931 (78.24%) of them were living outside of Beijing (Figure 2(A)), especially northeast China. More patients lived in urban areas (862, 72.44%) than those in the rural (304, 25.55%), leaving 24 (2.02%) patients without unclear habitats. Before the COVID-19 outbreak, 975 (81.93%) patients maintained regular follow-ups; most (693, 58.24%) visited PUMCH, 439 (36.89%) patients mainly visited local clinics, and the rest 4.87% presented vague answers. In terms of education level, 466 (39.16%) of the patients accomplished college, 678 (56.97%) obtained less educational qualifications, and the rest 46 (3.87%) refused to provide the education information (Table 1).

**Table 1.** Clinical characteristics of enrolled patients with kidney diseases.

Characteristics	Data (*N* = 1190)
Male, n (%)	620 (52.10)
Age (year, mean ± SD)	41.91 ± 15.56
Time since kidney biopsy [month, median, quantiles]	23.62 (3.72–40.19)
Location	
Beijing, n (%)	259 (21.76)
Outside of Beijing, n (%)	931 (78.24)
Habitat	
Urban, n (%)	862 (72.44)
Rural, n (%)	304 (25.55)
Unspecified, n (%)	24 (2.02)
Previous follow-up condition	
Regular follow-up, n (%)	975 (81.93)
Irregular follow-up, n (%)	215 (18.07)
Previous follow-up hospitals	
PUMCH, n (%)	693 (58.24)
Local clinics, n (%)	439 (36.89)
Unspecified, n (%)	58 (4.87)
Education levels	
Below high school, n (%)	412 (34.62)
High school, n (%)	266 (22.35)
Undergraduate or above, n (%)	466 (39.16)
Unspecified, n (%)	46 (3.87)
Comorbidities	
Diabetes mellitus, n (%)	180 (15.13)
Cardiovascular disease, n (%)	651 (54.71)
Hypertension, n (%)	639 (53.70)
Coronary artery disease, n (%)	22 (1.85)
Congestive heart failure, n (%)	13 (1.09)
Chronic respiratory disease, n (%)	8 (0.67)
Asthma, n (%)	4 (0.33)
Chronic obstructive pulmonary disease, n (%)	4 (0.33)
Pathological diagnoses	
Mesangial proliferative GN, n (%)	331 (27.82)
Membranous nephropathy, n (%)	317 (26.64)
Minimal change GN, n (%)	53 (4.45)
Focal segmental GN, n (%)	50 (4.20)
Other primary GN, n (%)	32 (2.69)
Tubular interstitial impairments, n (%)	68 (5.71)
GN secondary to immune diseases, n (%)	135 (11.34)
GN secondary to metabolic disorders, n (%)	97 (8.15)
Miscellaneous (amyloidosis, inherited, transplant, unclassified,etc.)	107 (8.99)
Immunosuppressive treatments	
Without immunosuppressive treatments, n (%)	508 (42.69)
Steroids only, n (%)	187 (15.71)
Immunosuppressants only, n (%)	91 (7.65)
Steroids and immunosuppressants, n (%)	255 (21.43)
Unspecified, n (%)	149 (12.52)
ESRD and initiated dialysis, n(%)	26 (2.18)

SD: standard deviation; COVID-19: coronavirus infection disease 2019; PUMCH: Peking Union Medical College Hospital. GN: glomerulonephritis. ESRD: end-stage renal disease. Unspecified meant the responder presented an unclear answer or refused to answer.

Primary glomerulonephritis was the most common pathological finding in our patients, taking up as many as 65.80%. Mesangial proliferative glomerulonephritis (27.82%) and membranous nephropathy (26.64%) ranked as the top two diagnoses. Among the secondary glomerulonephritis, diseases related to immune disorders (11.34%) were the most common cause, while metabolic disorders afflicted 8.15% of patients. Some chronic comorbidities were common in our patients, among which cardiovascular disease (54.71%), hypertension (53.70%), and diabetes mellitus (15.13%) were listed as the top three. Until our telephone interview, 44.79% of the patients were currently undergoing immunosuppressive treatments. Among all patients, 15.71% used steroids monotherapies, 7.65% used immunosuppressants without steroids, and 21.43% took the combination of both. Immunosuppressive treatments were not clearly reported among 12.52% responders. Unfortunately, 26 (2.18%) CKD patients developed into ESRD in the past three years, and all of them initiated dialysis before the COVID-19 outbreak (Table 1).

None of our patients was confirmed or suspected COVID-19. We excluded 26 dialysis patients for further investigation of follow-up conditions (Figure 1). Among the rest 1164 patients, 375 (32.22%) felt worried about COVID-19 due to their renal disease background. The infection outbreak significantly influenced the follow-up of these patients in various aspects. Regular face-to-face clinic visits were interrupted among 836 (71.83%) of them. Correspondingly, 693 (59.54%), 783 (67.27%), and 315 (27.06%) of our patients reported difficulties in laboratory examinations, medicine adjustments, and medicine purchases, respectively. None of them were forced to stop medications. In all patients, 255 (21.91%) utilized telemedicine methods, including internet consultation (122, 10.48%), instant message tools (62, 5.33%), telephone consultation (48, 4.12%), and email (23, 1.98%), in order to communicate with their previous doctors. Among the telemedicine users, 143 (56.08%) initiated the attempts after the COVID-19 outbreak. About 80% of telemedicine users were generally satisfied with the experiences (Table 2). The utilization of telemedicine was not relevant to patients’ sex (male vs. female, *p* = 0.742), age (<45-year-old vs. >45-year-old, *p* = 0.056), location (urban vs. rural, *p* = 0.281), education (college vs. below college, *p* = 0.859), nor attitude toward COVID-19 (worried vs. not worried, *p* = 0.643), but significantly relevant to the immunosuppressive treatments (*p* < 0.001). In patients receiving immunosuppressive treatments, 31.88% utilized a telemedicine experience, whereas only 17.12% of patients not receiving immunosuppression utilized telemedicine.

**Table 2.** The influence of COVID-19 pandemic on the management of patients with kidney diseases (excluded ESRD patients).

Influence	Total = 1164
Confirmed COVID-19, n (%)	0 (0.00)
Worried about COVID-19	375 (32.22)
Influence on face-to-face clinic visits	
Delayed or irregular, n (%)	836 (71.82)
No influence, n (%)	331 (28.44)
Unspecified, n (%)	23 (1.98)
Influence on laboratory examinations	
Delayed or irregular, n (%)	693 (59.54)
No influence, n (%)	469 (40.29)
Unspecified, n (%)	28 (2.41)
Influence on medicine adjustment	
Delayed or with difficulties, n (%)	783 (67.27)
No influence, n (%)	376 (32.30)
Unspecified, n (%)	30 (2.58)
Influence of medicine purchase	
Delayed or with difficulties, n (%)	315 (27.06)
No influence, n (%)	844 (72.51)
Unspecified, n (%)	31 (2.66)
Telemedicine choices, n (%)	255 (21.91)
Telephone consultation, n (%)	48 (4.12)
Instant message tools, n (%)	62 (5.33)
Internet consultation, n (%)	122 (10.48)
e-mail, n (%)	23 (1.98)
Initiation of telemedicine (among 255 users)	
Before COVID-19 outbreak	112 (43.92)
During COVID-19 outbreak	143 (56.08)
Feedback of telemedicine (among 255 users)	
Generally satisfied, n (%)	203 (79.61)
Not user-friendly, n (%)	29 (11.37)
Do not solve problems, n (%)	11 (4.31)
Relatively higher price, n (%)	6 (2.35)
Unspecified, n (%)	6 (2.35)

COVID-19: coronavirus infection disease 2019; Unspecified meant the responder presented unclear answer or refused to answer.

**Figure 3.**
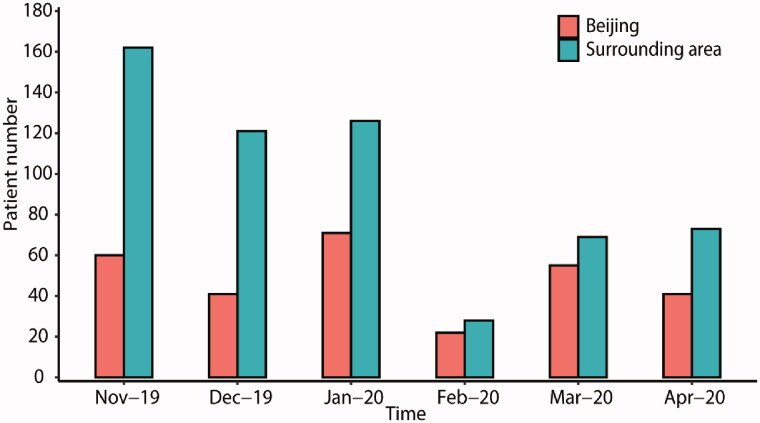
The temporal change of follow-up conditions in Beijing and surrounding areas. We compared the PUMCH face-to-face nephrology clinic visits each month (December 2019 to April 2020) to that of November 2019. Patients from Beijing and the surrounding provinces were analyzed. In February, the number of patients coming to the PUMCH nephrology clinic dropped to 36.67% and 17.28% for Beijing and the surrounding provinces, respectively. In March, follow-up visits gradually recovered back to 91.67% and 42.59% for them, respectively. PUMCH: Peking Union Medical College Hospital.

As illustrated in Figure 2(A), most of our patients came from northeast China, especially Beijing and its surrounding areas. The percentages of patients who reported difficulties in face-to-face clinic visits differed among provinces (Figure 2(B)). Over 60% of patients were affected mainly in Beijing and surrounding provinces, related to the severity of viral spread and strength of restriction measures [2]. By examining the most recent clinic visit date among patients from Beijing and its surrounding areas, the follow-ups were reduced to the minimum in February 2020 and slightly recovered in March and April, concordant with the tide and ebb of the COVID-19 situation in China. After the first transmission wave, face-to-face clinic visits among local Beijing CKD patients gradually increased to 55 follow-up visits in March 2020, recovering to 91.67% compared with the 60 visits in November 2019. However, the recovery of follow-up visits from surrounding areas was slower, adding up to 42.59% in March 2020 (69 visits), compared to November 2019 (162 visits) (Figure 3).

## Discussion

While the general population is susceptible to COVID-19, patients with kidney diseases are at risk of developing severe complications if infected [7–9]. More than 40% of inpatient COVID-19 cases might present with abnormal kidney function, and acute kidney injury was associated with increased mortality [7]. Based on current data, patients with existing kidney diseases should be watched during this pandemic.

To the best of our knowledge, this is the first study reporting the effects of COVID-19 on the outpatient follow-up of CKD patients. Patients who underwent renal biopsy within the last three years particularly need regular follow-up. These patients were typically undergoing medication tapering and close monitoring. Notably, steroids or immunosuppressants were the current medication among half of the participants. Reassuringly, none of the patients in our cohort was confirmed COVID-19. During the COVID-19 outbreak, most cities in China adopt stringent quarantine measures. We found in this investigation that one-third of our patients felt worried about the infection, and they might consequently adopt more delicate self-protection. This finding indicated that immunosuppressive treatments could be safely used in CKD patients during the COVID-19 pandemic when proper self-protection was utilized.

This pandemic profoundly influenced patient management in many ways. More than 70% of patients experienced interrupted face-to-face clinic visits. These routine clinic visits provided access to medications in at least 26% of the participants, who alternatively turned to local pharmacies or internet purchases. So far, we have not witnessed a visible short supply of these essential medications. Regular laboratory examinations and therapy adjustments were also affected in approximately 60% of them. Most patients adhered to the planned prescription, including relatively stable doses of antihypertensive drugs or procedures of steroid tapering. One-fifth of the patients turned to telemedicine, especially online consultation. The development of digital tools and set-up of virtual clinics may be hugely beneficial to our healthcare system [10], which could guarantee clinical care while minimizing physical gatherings in hospital. More involvement of physicians and patient education is needed to increase the efficacy of these tools further. Additionally, the utilization of online triage systems can prevent unnecessary hospital consultations and alleviate the workload of clinicians in the long term.

The COVID-19 outbreak also shaped the landscape of follow-ups and shifted it somehow toward telemedicine. In recent years, with the rapid development of modern communication technologies, telemedicine started to gain popularity among medical specialties [11–13]. Our patients were not intensive telemedicine users; however, our study indicated the necessity of promoting telemedicine, especially for patients who needed frequent monitoring and medicine adjustments. Patients from remote areas could benefit from telemedicine to overcome inconvenience. In our study, patients who attempted telemedicine during the quarantine period contributed more than half of the total users.

Some progressive providers, including PUMCH, have already realized the upcoming challenge and initiated online medical service trials in China. In the early stage of the COVID-19 outbreak, PUMCH rapidly announced the online medical services, starting from fever counseling, then gradually expanded to 49 departments participated with >800 physicians and pharmacists. This system has so far benefited more than 50,000 patients. Such an online system may dramatically affect medical service in regional China.

Though we described the real-world follow-up situation during the COVID-19 pandemic in various aspects, there are several limitations to this study. First, this is a single-center observation; we should consider regional diversities and disease spectrum when interpreting the findings. The mainstream follow-up approaches could be different in other countries. Second, the risk of COVID-19 in patients with kidney disease cannot be deduced from this study. But our data suggest that proper protection measures could mitigate the risk. Third, our data are less representative of the patients from epicenters, such as Wuhan and its surrounding areas. Though the severity of viral spread was less severe in our coverage areas, the influence of quarantine measures was demonstrated. Fourth, it is not trivial to include CKD stages when addressing CKD follow-up conditions since patients with higher stages usually need closer observation. However, the inquiries of serum creatinine levels were not reliable through telephone visits.

Recently, China has gradually eased restriction measures. However, the COVID-19 transmission persists, and waves of COVID-19 may recur. More than 150 countries are currently experiencing different viral spread phases, coupled with various control measures. Elaborate patient management still requires further research from different perspectives.

## Conclusion

In this current study, we investigated the follow-up conditions during the COVID-19 pandemic in a relatively large number of CKD patients. We found the risk of COVID-19 was mitigated in patients with kidney disease and other co-existing risk factors when proper protection was utilized. The routine medical care was disrupted during the pandemic, and telemedicine could be a reasonable alternative method. We hope our experience could provide insight for other hospitals when the face-to-face consultation for reviewing patients is not feasible. Future studies are encouraged to provide more robust evidence about the prognosis of some certain chronic diseases using telemedicine.

## Data Availability

The authors presented all the necessary data as tables and figures in the manuscript. Related information is accessible under request to the corresponding author.
